# Current and Novel Emerging Medical Therapies for Peripheral Artery Disease: A Literature Review

**DOI:** 10.34172/apb.2023.025

**Published:** 2022-04-04

**Authors:** AmirAhmad Arabzadeh, Elnaz Faghfuri, Saiedeh Razi Soofiyani, Elaheh Dalir Abdolahinia, Samaneh Siapush, Kazem Nejati-Koshki, Bita Shahrami, Vahid Asghariazar, Yasamin Pahlavan

**Affiliations:** ^1^Department of Surgery, School of Medicine, Ardabil University of Medical Sciences, Ardabil, Iran.; ^2^Digestive Disease Research Center, Ardabil University of Medical Sciences, Ardabil, Iran.; ^3^Department of Molecular Medicine, Faculty of Advanced Medical Sciences, Tabriz University of Medical Sciences, Tabriz, Iran.; ^4^Research Center for Pharmaceutical Nanotechnology, Biomedicine Institute, Tabriz University of Medical Sciences, Tabriz, Iran.; ^5^Department of Medical Biotechnology, Faculty of Advanced Medical Sciences, Tabriz University of Medical Sciences, Tabriz, Iran.; ^6^Pharmaceutical Sciences Research Center, Ardabil University of Medical Sciences, Ardabil, Iran.; ^7^Department of Clinical Pharmacy, Tehran University of Medical Sciences, Tehran, Iran.; ^8^Immunology Research Center, Ardabil University of Medical Sciences, Ardabil, Iran.; ^9^Biosensor Sciences and Technologies Research Center, Ardabil University of Medical Sciences, Ardabil, Iran.

**Keywords:** Peripheral artery disease (PAD), Angiogenesis, Nano-therapy, Cell therapy, Gene therapy

## Abstract

Despite the improvements in endovascular techniques during the last decades, there is still an increase in the prevalence of peripheral artery disease (PAD) with limited practical treatment, which timeline impact of any intervention for critical limb ischemia (CLI) is poor. Most common treatments are not suitable for many patients due to their underlying diseases, including aging and diabetes. On the one hand, there are limitations for current therapies due to the contraindications of some individuals, and on the other hand, there are many side effects caused by common medications, for instance, anticoagulants. Therefore, novel treatment strategies like regenerative medicine, cell-based therapies, Nano-therapy, gene therapy, and targeted therapy, besides other traditional drugs combination therapy for PAD, are newly considered promising therapy. Genetic material encoding for specific proteins concludes with a potential future for developed treatments. Novel approaches for therapeutic angiogenesis directly used the angiogenetic factors originating from key biomolecules such as genes, proteins, or cell-based therapy to induce blood vessel formation in adult tissues to initiate the recovery process in the ischemic limb. As PAD is associated with high mortality and morbidity of patients causing disability, considering the limited treatment choices for these patients, developing new treatment strategies to prevent PAD progression and extending life expectancy, and preventing threatening complications is urgently needed. This review aims to introduce the current and the novel strategies for PAD treatment that lead to new challenges for relief the patient’s suffered from the disorder.

## Introduction

 Peripheral artery disease (PAD) is characterized by a pathogenic condition with ankle-brachial index lower or equal to 0.90 with asymptomatic or concomitant by symptoms, including the atypical leg pain, alternative claudication, critical upper and lower acute or chronic limb ischemia, ischemic pain, and ulcerations and atherosclerosis in arteries, atherosclerosis, amputation, stroke and myocardial infarction (MI). According to recent reports, 237 million of 25 years old and older people were suffered from PAD in the last decade. Chronic limb-threatening ischemia, defined as the most violent form of PAD, originated from narrowing or occlusion of arteries, and there is a significant risk of limb amputation, mortality. It estimated costs to the healthcare system to range from $84 billion to $380 billion annually.^1^ PAD impacts on the quality of life and functional capacity cardiovascular risk of individuals.^2^ The prevalence of PAD sharply rises with age; almost 20% of the US people in the mead of their eighty suffer the most from PAD.^3^ The presence of a stenosis/occlusion of intra-renal arteries is the PAD characteristic.^4^ Epidemiologic studies shed light on the global effect of these disorders, suggesting an extreme increase in PAD prevalence in low and middle-income countries, supporting that we will confront a universal PAD pandemic that affects more than 200 million populations in both well-developed and developing countries.^5^

**Figure 1:**
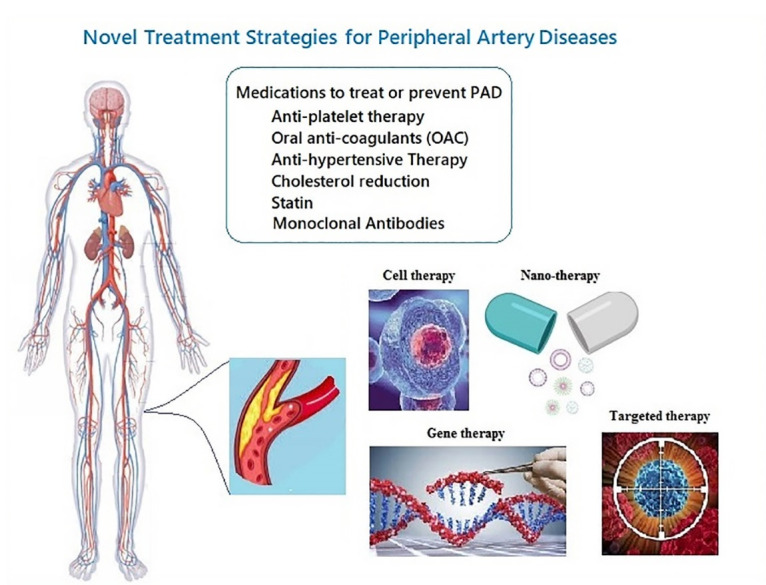
Novel emerging medical therapies for peripheral artery disease.

 Recently, studies on molecular mechanisms of disease have attracted the attention of researchers. Mechanisms like inflammation, small molecules, signaling pathways, autoimmunity, and apoptosis with the pathogenesis of diseases introduce novel molecules for targeting as a therapeutic promise.^6-9^ Current common therapies for PAD patients consist of treatment strategies, including lifestyle modifications, exercise, smoking cessation, and pharmacologic treatments like antiplatelet therapy, anticoagulation, management of blood pressure, cholesterol reduction, and peripheral vasodilators, endovascular repair, and surgery.^10-14^ The common side effects have reported after administrating medications for PAD are from severe hemorrhages in some cases to gastrointestinal dysfunction, major bleeding events, osteoporosis, thrombocytopenia, hypersensitivity reaction and skin necrosis^15,16^ Difficulty in controlling the lifestyle in different classes of society on the one hand and the detrimental side effects of common medications, such as bleeding, on the other hand, emphasize the need for the emergence of new, advanced, and accurate therapies. Moreover, due to the high rate of mortality and morbidity that lead to the disability of individuals and considering the limited treatment choices for patients suffering from severe PAD, developing new treatment strategies to prevent the disease progression and control the life-threatening complications is urgently needed. Nowadays, the promising role of regenerative medicine, cell therapy, targeted therapy, gene therapy, Nano-therapy, and combination drug therapies in PAD management is highlighted (Figure 1). In patients who suffer from severe atherosclerotic disease, administration of the cell populations with the potential of activating an angiogenic pathway may lead to the neo-vessels formation and improve the perfusion in the affected limb.^17^ This review aims to introduce the current and novel strategies around PAD leading to new challenges, and global debate around for treatment of patients suffering from this classification of diseases focuses on translational research.

## Pharmacologic therapy

###  Antiplatelet therapy

####  Antiplatelet monotherapy

 The antiplatelet agents in PAD include aspirin, clopidogrel, ticagrelor, and vorapaxar. The effectiveness of aspirin in symptomatic PAD is well established. However, the impact of optimum doses of this agent in PAD is still unclear and may be determined in further trials.^18-21^ A randomized, blinded, double-dummy trial comparing ticagrelor versus clopidogrel in 135 885 patients with PAD showed no significant difference between them at the endpoint of cardiovascular mortality, MI, or ischemic stroke.^22^

## Dual antiplatelet therapy (DAPT)

 Although the role of DAPT in acute MI was well established, the impact of DAPT in PAD is unclear. Researchers conducted an observational cohort study of 629 patients with claudication or critical limb ischemia (CLI) treated either with DAPT or aspirin monotherapy and followed them for 3 years. There was a significant reduction in major adverse cardiovascular events (MACE) in patients receiving DAPT vs. aspirin alone.^23^ Recently, some clinical trials focus on the optimizing duration of DAPT following revascularization. The ASPIRE (Antiplatelet Strategy for Peripheral Arterial Interventions for Revascularization of Lower Extremities) discusses the effect of clopidogrel on the background of low-dose aspirin therapy for a clinically demonstrated duration compared to 12 months in patients receiving endovasculartherapy.^24^ The LONGDAPTPAD (Effect of Prolonged DAPT after Lower Extremity Percutaneous Transluminal Angioplasty in Patients with LE-PAD) compares 3 months DAPT compared with 12 months.^25^ In addition to chemical drugs, supportive therapies to relieve pain are common in patients. The mechanism of the action of traditional medicines on pain has been previously investigated.^26,27^

###  Oral anticoagulant (OAC)

 Rivaroxaban is a direct OAC that inhibits Factor Xa. Although it is a relatively new medication, it was widely used to treat venous thromboembolism and stroke prevention in non-valvar atrial fibrillation. In a double-blind trial of 27 395 participants with stable atherosclerotic vascular disease, those given Rivaroxaban plus aspirin had better cardiovascular outcomes and the most severe bleeding events than those given aspirin alone. Not only Rivaroxaban monotherapy does not lead to better cardiovascular outcomes than aspirin monotherapy but also it resulted in the most severe bleeding events.^28^ This combination therapy signifies an important breakthrough in the treatment of patients with PAD.^29^ In a double-blind trial, patients with PAD undergoing revascularization were randomized to take Rivaroxaban plus aspirin or placebo plus aspirin. Compared to aspirin monotherapy, this combination therapy significantly reduced the composite outcome of acute limb ischemia (ALI), major amputation for vascular causes, MI, ischemic stroke, or cardiovascular causes-related death.^30^

###  Anti-hypertensive therapy

####  Angiotensin-converting enzyme inhibitors (ACEI)/Angiotensin receptor blockers (ARB)

 Administration of ACEI/ARBs is associated with reducing MACE as well as death rates in patients with CLI. There was no correlation between ACEI or ARB administration and limb-related outcomes. Paying more attention to secondary prevention measures in CLI patients can significantly improve long-term outcomes.^31^

###  Cholesterol reduction agents 

####  Statin therapy 

 Among the most suitable effects of statins in PAD are decreased platelet activation, endothelial dysfunction, and inflammatory responses to the atherothrombotic progression. Statins cause beneficial changes in atherosclerotic plaque composition, reducing their necrotic core and overall atheroma volume. Initiation of statin therapy in patients with the peripheral arterial occlusive disease after index revascularization is effective and safe with an effect size comparable to previous studies.^32,33^

###  Monoclonal antibodies

 Evolocumab reduces the low-density lipoprotein (LDL)-Cholesterol plasma levels by inhibiting the function of proprotein convertase subtilisin-Kexin type 9 (PCSK9). A combination of evolocumab and statins was compared with placebo plus statins in 27 564 patients with cardiovascular disease in a double-blind, randomized trial. This study noted a 15% reduction in composite cardiovascular events at 2.2 years for evolocumab-treated patients, with a low frequency of side effects for both groups.^34^

 Patients with PAD are at high possibility of cardiovascular events, and inhibition of PCSK9 with evolocumab significantly reduces this risk. Besides, lowering LDL cholesterol by evolocumab reduces the risk of major adverse limb events.^35^

## Gene therapy and targeted therapy

**Figure 2:**
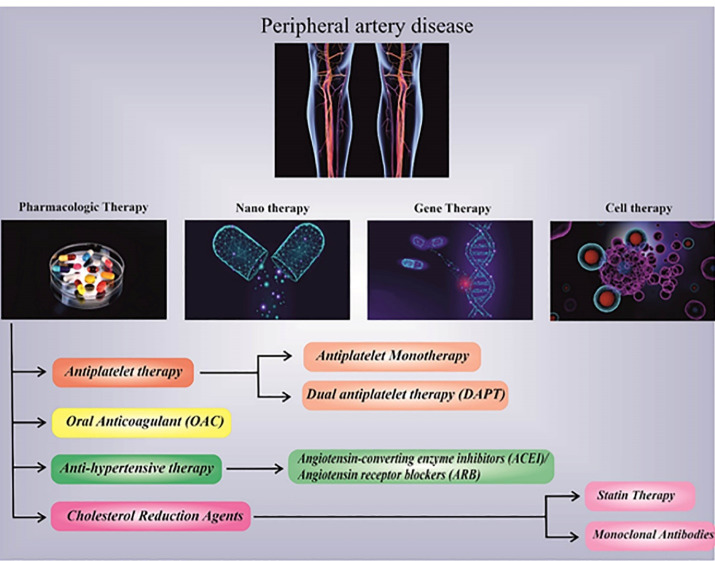
Advanced treatment strategies include regenerative medicine, cell-based therapies, Nano-therapy, gene therapy, and targeted therapy, besides other traditional drugs combination therapy introduced for PAD.

 While other medications and treatments are not suitable for many patients due to their other diseases such as aging and diabetes, gene therapy and targeted therapy remain promising therapy in which genetic material encoding for specific proteins leads to potential future treatments.^7^ This is called therapeutic angiogenesis, which means directly using angiogenesis factors such as genes, proteins, or cell-based therapy to induce blood vessel formation in adult tissues and initiate the recovery process in the ischemic limb (Figure 2). Hence, the efficiency of therapeutic angiogenesis would increase if the treat directly administered into ischemic muscle tissues via intra-arterial or intramuscular (IM) routes.^36^ We must consider that finding the best route to deliver the gene’s carrier is as important as the type of vectors because the effectiveness of gene transfer would reduce while using naked plasmid or adenovirus vectors injected via the vein. Although plasmids have very low efficiency and a short duration of transduction, they are still safe and easy to manufacture.

 Furthermore, some cells can take up spontaneously naked plasmid DNA.^37^ Moreover, vectors-mediated gene transfer of proangiogenic factors, vascular endothelial growth factor (VEGF), fibroblast growth factor, and hepatocyte growth factor (HGF), for instance, would cause hemangioma formation.^38^ Therefore, the long-expression of proangiogenic factors should be avoided when finding effective treatment options.^39^ While several studies used naked plasmid DNA expressing HGH and VEGF, researchers decided to use internal ribosome plasmid vectors in patients with PAD to examine its safety and efficacy.^40^ Their data confirm that IM administration of internal ribosome carried VEGF165/HGF is safe, feasible, and effective for patients who suffer from ischemia in the limb body. Using a lentivirus-mediated vector, others investigate the expression of endothelial Per-Arnt-Sim domain protein 1 (EPAS1) on PAD in a rat model. The hypoxia-inducible factors-2α or originally named EPAS1 associated with pathologic vascular wall remodeling, and identifying the HIF pathway is very important in the line of introducing novel therapeutic strategies.^41^ Recent studies revealed that mRNA expressions of EPAS1, HGF, basic fibroblast growth factor (bFGF), and VEGF were up-regulated in the lenti-EPAS1-treated sites resulted in recovered limb function and circulation after 7 days.^42^ In addition, during tissue ischemia in PDA, lipid oxidation is associated with inflammation, leading to the protein adducts inducing angiogenesis. Recent reports indicated that CEP (x-[2-carboxyethyl] pyrrole) as one of these proteins has a profound positive effect on angiogenesis.^43^ CEP (x-[2-carboxyethyl] pyrrole) protein, a product of lipid oxidation, induces angiogenesis in PAD.^38^

 To identify novel therapeutic targets for gene therapy and any other specific new techniques, in silico analysis was conducted by researchers to identify the molecular basis of PAD. They found a significant enhancement in the inflammatory response, immune response, chemokine-mediated signaling pathway, and JAK-STAT signaling pathway. Interleukin 6 (IL-6), C-X-C Motif chemokine ligand 12 (CXCL12), interleukin 1 beta (IL1B) are some of the chemokines that participate in the progression of the disease. IL6, CXCL12, IL1B are some of the chemokines that participate in the progression of the disease, and signal transducer and activator of transcription 3 were differentially expressed and regulated 27 potential target miRNAs in PAD. Although this data needs to be further verified in experimental studies, this makes a new vision regarding new targets for gene therapy. This indicates that gene therapy should be considered an extra tool for PAD treatment, while there is room for another technical improvement. On the other side, one problem with gene therapy is the fact that it is strongly linked to dose-dependent microvascular permeability. Around half of the patients faced moderate or severe edema during clinical trials, indicating that long-term and slow-release are more effective. This is the main reason that some experts suggest cell therapy instead of gene therapy, while its effectiveness is still the place of debate. During PAD and the subsequence, CLI as its end-stage redox biology and oxidative stress play an important role in this setting. Hence, some of the studies pay attention to strategies that inhibit ischemia through pharmacological intervention, such as statins, angiotensin-converting enzyme inhibitors, and phosphodiesterase inhibition. Numerous studies provided us encouraging data about the potential role of several agents, but in targeted therapy, underlying conditions that may contribute to an improvement of PAD or failure in treatment must be considered.^29^

 Although the use of gene therapy to treat apple disease is an attractive and promising method, it is also associated with the general limitations of gene therapy methods. In gene therapy methods, the use of silencers is one of the most common methods of gene therapy. Proper design, fabrication, transmission, stability, and targeting of silencers are some of the major challenges facing gene therapy methods. For some of these issues, solutions have been proposed however are not currently definitively accepted. For example, the development in altered bases that are created to increase the stability of silencers such as siRNA within the body system. Other limitations of gene therapy are the development of immunological responses and innate immune system stimulation that can lead to unwanted and severe symptoms. Another challenge in the treatment of various diseases with gene therapy is Accelerated blood clearance, which leads to the rapid elimination of gene therapy cases from the bloodstream.

## Regenerative medicine and cell therapy

 Cell therapy is known to be curative for disease conditions like diabetes mellitus, vascular complications, ischemic stroke, and trauma which offered novel treatment options for regenerative medicine, including the engineering and regenerating de novo generated cells considered the emerging field of medicine and helped substitute current treatment with newly developed treatment strategies. The beneficial focus on cell therapy for PAD is not limited to the injection of cells into the vascular network to effectuate a medical impact. Cell therapy could activate unknown cytoprotective/ regenerative pathways to recover limb activity via its independent effect on the neo-vessel formation.^44^

 Experimental studies have been used the hindlimb ischemia models to investigate the effectiveness of cell therapy in angiogenesis promoting and reducing skeletal muscle damages.^45^ Alternatively, most clinical studies reported that the cell transplantation effectiveness in patients suffers from CLI.^46,47^

 Identifying ideal endothelial progenitor cells’ source for therapeutic application in PAD needs further investigations. Cell therapy projects using various cell types are investigated in different phases of preclinical/clinical trials for PAD treatment. In these studies, each cell type indicated a promising approach for PAD treatment in preclinical studies. Several randomized, controlled trials were carried out in this field from 2010, which are discussed below (Table 1).

**Table 1:** Randomized controlled trials of stem cells for critical limb ischemia

**N**	**Cell type(s)**	**Route**	**Outcomes associated with treatment**	**Ref**
41	BMCs	IM/ IA	Improved TcO2, pain scale, EQ5D and significant reduction in the Rutherford category of CLI (no differences among functional parameters in patients undergoing IM versus IA delivery	^48^
28	BM-MSC	IM	Increase in ABPI & ankle pressure, improved rest pain	^49^
81	BMSC	sham injection/ IA	Reduction in ulcer size and improvement in pain-free walking distance	^50^
62	BMCs	local IM/IA	Improved TcpO2, pain scale, quality of life, wound healing, IA and Rutherford category	^51^
48	BMAC	IM	Improved pain rest	^52^
40	G-CSF mobilized peripheral blood (PBMNCs)	SQ	Improved pain score, amputation rates, collateral vessel development, and number of healed limb ulcers	^53^

BMCs, bone marrow cells; TcO2, total carbon dioxide; TRC, tissue repair cell; EQ5D, quality-of-life questionnaire; CLI, critical limb ischemia; IA, intra-arterial; IM, intramuscular; BM-MSC, Bone marrow derived mesenchymal stem cells; ABPI, ankle brachial pressure index; PBMNC, peripheral blood mononuclear cells; GCSF, granulocyte colony-stimulating factor; SQ, subcutaneous injection.

 Mesenchymal stem cells (MSCs) are promising as a treatment for peripheral arterial diseases. MSCs can be derived from a variety of potential sources and administered by several techniques.^54^ Intramuscular (IM) injection has been the most common method used in preclinical and clinical studies. MSCs have also been injected intravenously in both preclinical and patient models. In addition, several clinical trials performed on bone marrow-derived mononuclear cells have successfully used intra-arterial injections. Some investigations have shown that MSCs are therapeutically effective when injected immediately after induction of hind limb ischemia. In a similar study, a 24-hour delay after ischemia induction has also been shown to be more effective than immediate injection. The delay between the onset of disease and therapeutically effective administration of MSCs seems to be wide. For example, bone marrow-derived MSCs transplanted one hour, one week, or two weeks after MI were all effective, although injection approximately one week after infarction showed the highest recovery of myocardial function as the acute inflammatory reaction is almost complete by this time, the success in delayed injections may be a consequence of enhancement in the survival of transplanted cells. More concern is about clarifying the optimal dose for cell therapy because in general, only a small fraction of MSCs survives and can exert a therapeutic effect through paracrine mechanisms or final determination. The tendency to use large numbers of MSCs for transplantation has to be weighed against reports of complications from such efforts. However, further research is needed to optimize the concentration, timing and delivery of MSCs that are critical for effective neovascularization in PAD^55^ as described in Table 1.

 There are many challenges to using human embryonic stem cells, as obtaining these cells requires the destruction of ballast cells, which is not accepted by some ethical and religious schools. To solve this problem, the use of other types of stem cells such as adult stem cells, amniotic stem cells, and induced pluripotent stem cells was suggested. It can be said that the most important limitations related to cell therapy are technical limitations about obtaining, differentiating, and transferring these cells to the intended destination.^56,57^

## Nano-therapy

 Nono-technology and nanoparticles (NPs) are important strategies that can help clinicians deliver the drug agents to the specific site, finding the new route of administration and constant treatment of diseases. Over the previous century, nucleic acids and protein distribution have been widely used in scientific and clinical trials to increase the effectiveness of PAD therapy.^58^ However, due to these molecules’ short distribution half-life, applying these naked molecules has not resulted in an appropriate clinical effect. Different forms of NPs were produced to address this problem and are being used to transport nucleic acids and proteins.^59-61^

 Dendrimers were used as carriers for small interfering RNA (siRNA) and complementary DNA (cDNA) plasmid distribution due to their exceptional characteristics, including strongly branched polymers with three major regions (the nucleus, branching zone, and branch extremities) where alteration can happen. This structure was studied in the treatment of coronary artery disease and gene transfer to dysfunctional smooth muscle cells (SMCs) in rabbit vessels. It has strong effectiveness and therapeutic potential for PAD.^62-64^ In addition, these nanostructures are commonly known to be effective in transduction due to electrostatic interactions between the primary amine terminal groups with incorporated DNA or siRNA.^65-67^ Liposomes are often used as vascular transmission vectors because of their biological compatibility and wide range of vascular uses.^68,69^ Proteins are encapsulated within NPs to shield them from enzyme degradation and ensure regulated release into cells used for greater therapeutic efficacy. These carriers can help improve drug reaction by targeting and adhering to the affected areas, thus synergizing the drug’s effectiveness.^40,70^ By increasing permeability and retention, nanoliposomes can be useful in treating myocardial ischemia by improving angiogenesis and treating ischemic myocardium as a carrier of therapeutic agents.^71^ Researchers improved liposomal blood supply and promote drug transfer by altering the surface of liposomes with polyethylene glycol (PEG) (Figure 1).^72^

 For PAD therapy, cyclic arginylglycylasparticacid (RGD) peptide conjugated liposomes were engineered to interact with P-selectin and integrin receptors.^73,74^ Polymer NPs have been used to transfer genes for targeted ischemic treatment in peripheral arteries and may be used to substitute viral vectors and fix their safety issues. Loading cDNA VEGF on NP compared to naked plasmid can stimulate angiogenesis in rabbit ischemic models. NPs containing chitosan, thioglycolic acid, and encapsulated the secretoneurin peptide to improve angiogenesis in the hind leg of mice, resulting in controlled release for optimum effects and dense arteriole formation. Probucol is an anti-angiogenesis and apoptosis-inducing agent used to control hypercholesterolemia. The drug was synthesized in PLGA polymer carriers and liposomes. According to the findings, probucol in PLGA nanocarrier successfully prevented seizures through injection into rabbit arteries following angioplasty. Growth factors in combination with NPs have emerged as a promising strategy for treating patients with PAD. bFGF can be used as long-term angiogenic therapy in heparin-bound poly(L-lactide-co-glycolide) nanospheres (HCPNs). This compound was studied to treat a mouse ischemic limbs model, in which microvasculature developed in ischemic organs treated with bFGF delivery, and it was discovered that sustained release of bFGF from HCPN could enhance the angiogenic effect.^75^

**Figure 3:**
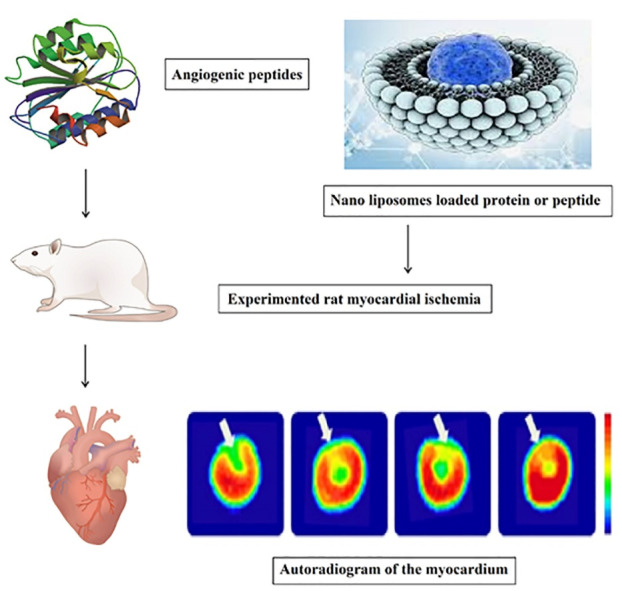
The therapeutic effects of angiogenic peptides loaded in PEGylated Nano liposomes improve perfusion defects. Reperfusion was administered after 30 minutes of rat myocardial ischemia followed by treatment with angiogenic peptides.

 According to recent report, angiogenic peptide-loaded Nano liposomes improve vascular density. The perfusion depicts an autoradiogram of the myocardium after 30 minutes of ischemia and reperfusion. To detect the result of therapy, tetrofosmin ^99m^Tc was used, which demonstrated a reduction in drug activity. The therapeutic effects of angiogenic peptides loaded in PEGylated nano liposomes improve perfusion defects. (Figure 3).^72^

## Conclusion

 The beneficial effects of PAD cell therapy are not limited to the cells in the vascular network; the paracrine effects induced by the secretion of antigenic mediators are so important. During PAD and the subsequence, CLI as its end-stage redox biology and oxidative stress play an important role in this setting. Hence, some of the studies pay attention to strategies that inhibit ischemia through pharmacological intervention, such as statins, angiotensin-converting enzyme inhibitors, and phosphodiesterase inhibition. Numerous studies provided encouraging data about the potential role of several agents, but in targeted therapy, underlying conditions that may contribute to an improvement of PAD or failure in treatment must be considered. Scientific researchers have indicated that novel treatment strategies like regenerative medicine, cryotherapy provides the growth of new healthy tissues, safe and effective autologous therapy, fast recovery of patients from injuries, and therapeutic surgeries, and that’s benefits consist for many years. Gene and targeted therapy can resolve and eliminate the compared problems related to the side effects of current drug treatments like anticoagulants, including bleeding, abdominal pain, flatulence, headache, lethargy, dizziness, and fever. Novel emerging treatment strategies for PAD may be a bright prospect to replace new and practical treatments with fewer side effects besides new, more efficient, and comfortable treatment options for patients and clinicians to advance clinical decisions.

## Acknowledgments

 The authors thank Ardabil University of Medical Sciences, Deputy of Research and Technology, for their collaboration with this review.

## Competing Interests

 The authors declare no conflict of interest.

## Ethical Approval

 Not applicable.
